# Early Transcriptional Response to Monensin in Sensitive and Resistant Strains of *Eimeria tenella*

**DOI:** 10.3389/fmicb.2022.934153

**Published:** 2022-07-04

**Authors:** Hongtao Zhang, Lei Zhang, Hongbin Si, Xianyong Liu, Xun Suo, Dandan Hu

**Affiliations:** ^1^College of Animal Science and Technology, Guangxi University, Nanning, China; ^2^Key Laboratory of Animal Epidemiology and Zoonosis of Ministry of Agriculture, National Animal Protozoa Laboratory, College of Veterinary Medicine, China Agricultural University, Beijing, China

**Keywords:** *Eimeria tenella*, monensin, drug resistance, RNA-seq, *in vitro*

## Abstract

*Eimeria* parasites are the causative agents of coccidiosis, a common parasitic disease in poultry and livestock that causes significant economic losses to the animal husbandry industry. Ionophore coccidiostats, such as monensin and salinomycin, are widely used for prophylaxis of coccidiosis in poultry. Unfortunately, widespread drug resistance has compromised their efficacy. As a result, there is an increasing need to understand the targets and resistance mechanisms to anticoccidials. However, how *Eimeria* parasite genes respond to ionophores remains unclear. In this study, resistance to monensin was induced in *E. tenella* through serial generations of selection. Both sensitive and resistant *E. tenella* sporozoites were treated with 5 μg/ml monensin for 0, 2, and 4 h, respectively. Gene transcription profiles were then compared by high-throughput sequencing. The results showed that protein translation-related genes were significantly downregulated after drug induction. A total of 1,848 DEGs were detected in the sensitive strain after 2 h of exposure, whereas only 31 were detected in the resistant strain. Among these DEGs in the sensitive strain, genes associated with protein degradation were significantly upregulated, supporting the autophagy-like parasite killing theory. Then, 4 h of exposure resulted in additional 626 and 621 DEGs for sensitive and resistant strains, respectively. This result implies that the gene transcription in sensitive strain is more susceptible to monensin treatment. Our results provide gene expression landscapes of *E. tenella* following monensin treatment. These data will contribute to a better understanding of the mechanism of drug resistance to polyether ionophores in coccidia.

## Introduction

The intestinal disease coccidiosis, caused by protozoan parasites of the *Eimeria* species, is one of the most important diseases in poultry and livestock industry (Dubey, 2019). According to the latest estimates, chicken coccidiosis costs more than £10 billion annually (Blake et al., 2020). To control coccidiosis in poultry, anticoccidial drugs (e.g., monensin, salinomycin, diclazuril, etc.) are extensively used for chemoprophylaxis (Kadykalo et al., 2018), which has led to an on-board drug resistance of the parasite (Peek and Landman, 2011). Monensin, a highly effective polyether ionophore coccidiostat, has been used commercially for more than 50 years to control coccidiosis in chickens (Chapman et al., 2010). Despite the fact that monensin-resistant *Eimeria* parasites were widely reported soon after their introduction (Jeffers, 1974, 1978; Augustine et al., 1987; Djemai et al., 2016), monensin is still extensively used in the poultry industry. Thus, there is an urgent need to clarify the mode of action and the mechanism of drug resistance in *Eimeria*.

Previous studies on the mode of action of monensin in *E. tenella* have shown that the activity of Na^+^-K^+^ ATPase increases after monensin treatment, leading to lactate accumulation and ATP depletion (Smith and Galloway, 1983). Consequently, a net influx of Na^+^ into the parasite was suggested, which would lead to osmotic swelling and eventually to parasite bursts (Smith et al., 1980; Smith and Galloway, 1983). Besides, studies have also shown that the outer membrane of sporozoite is structurally altered after exposure to monensin (Wang et al., 2006; Del Cacho et al., 2007), and that autophagy may be involved in monensin-mediated killing of *E. tenella* (Qi et al., 2020). The current series of studies by Arrizabalaga's group has been highly successful in dissecting the mode of action and mechanism of resistance of monensin in *Toxoplasma gondii*. They reported that disruption of a mitochondrial MutS DNA repair enzyme, TgMSH-1, coffers monensin resistant to the parasite (Garrison and Arrizabalaga, 2009). They also found that monensin causes TgMSH-1-dependent late-S-phase cell cycle arrest (Lavine and Arrizabalaga, 2011) and autophagy-like death in *T. gondii* (Lavine and Arrizabalaga, 2012). The autophagy can be indicated by the translocation of ATG8 to the autophagosomes, and the monensin-exposed parasites can be rescued by autophagy inhibitor 3-methyladenine (Lavine and Arrizabalaga, 2012). Monensin treatment also resulted in mitochondrial alterations in *T. gondii*, including reduced mitochondrial membrane potential and morphological changes (Lavine and Arrizabalaga, 2012; Charvat and Arrizabalaga, 2016). These deleterious effects could be mitigated by antioxidants or overexpression of antioxidant proteins, suggesting an oxidative stress induced by monensin in *T. gondii* (Charvat and Arrizabalaga, 2016).

An in-depth study on parasite responds to monensin treatment is essential for clarifying the mode of action. Lavine and Arrizabalaga (Lavine and Arrizabalaga, 2011) highlighted that canonical histones were significantly upregulated in *T. gondii* after 24 h of exposure to monensin, suggesting that monensin alters the cell cycle of the parasite. More recently, Zhai et al. (2020) showed that protein biosynthesis-related pathways were significantly downregulated in *T. gondii* after monensin treatment. Quantitative proteomic analysis was also used to understand the differences in gene expression between *E. tenella*-sensitive and -resistant strains (Thabet et al., 2017). However, the different genetic backgrounds of the parasites may introduce noise into the results. Herein, we present early transcriptional profiles of *E. tenella*-resistant and its sensitive parental strain in response to monensin treatment, to better understand the mode of action and molecular mechanisms of drug resistance phenomenon.

## Materials and Methods

### Ethical Statement

The use of animals in this study was approved by the Administration Committee of Laboratory Animals in Guangxi University and was performed in accordance with the Institutional Animal Care and Use Committee guidelines (Approval No: Gxu-2021-013).

### Animals and Parasites

The 2–4-week-old San Huang broilers purchased from a local company were used for passaging. All birds were fed a coccidia-free diet and water *ad libitum*. The monensin-resistant Houghton strain (ETH-R) was generated by 16 generations of serial passage under gradient monensin treatment (from 50 to 250 ppm) using the *E. tenella* Houghton (ETH-S) strain as the parental strain. ETH-S was sensitive to monensin treatment at 100 ppm and the resistant strain ETH-R was susceptible to treatment at 250 ppm. Detailed procedures for resistant strain generation and genetic trajectory alteration will be published soon.

### Sporozoites Purification and Monensin Treatment

ETH-S and ETH-R unsporulated oocysts were harvested after 9 days post-infection of six cages of chicken independently and sporulated in 2.5% K_2_CrO_4_ for 48 h, the sporozoites of both ETH-S and ETH-R were purified by Percoll density gradient method within 1 week as described previously (Dulski and Turner, 1988). Briefly, sporocysts were recovered from sporulated oocysts by a 50% Percoll density gradient after glass-dead grinding. The sporocysts were then excysted in excystation buffer at 42°C for 60 min. The sporozoites were purified by another gradient with 55% Percoll after excystation. The viability of sporozoites was tested before *in vitro* culture by trypan blue staining, and only sporozoites with >95% viability were used. For the monensin treatment, about 1.5 × 10^7^ fresh sporozoites of ETH-S and ETH-R strains were cultured in DMEM containing 5 μg/ml monensin for 0, 2, and 4 h, respectively. They were named S0, S2, S4, R0, R2, and R4. The number represents the time of exposure and the letters “S” and “R” stand for ETH-S and ETH-R strains, respectively. Finally, all samples were washed two times with ice-cold PBS and immediately stored at −80°C in TRIzol (Invitrogen, Beijing, China). Each treatment consisted of two or three biological replicates.

### RNA Extraction, Library Preparation, and RNA-Seq

Total RNAs were isolated using TRIzol regent and genomic DNA was removed by DNase I (Tiangen Biotech Co., Ltd, Beijing, China). The purity, concentration, and integrity of RNAs were tested using NanoPhotometer® (IMPLEN, CA, USA), Qubit® RNA Assay Kit in Qubit® 2.0 Fluorometer (Life Technologies, CA, USA), and RNA Nano 6000 Assay Kit of the Bioanalyzer 2100 system (Agilent Technologies, CA, USA), respectively. Only qualified samples were used for library preparation. Sequencing libraries were generated using the TruseqTM RNA Sample Prep Kit (Illumina, CA, USA) according to the manufacturer's recommendations. Sequencing was performed using the Illumina Hiseq TM platform to generate 150 bp paired-end reads. The original sequencing data could be found in the Sequence Read Archive database under the accession number PRJNA832043.

### Bioinformatics

Paired-end clean reads were aligned to our newly generated reference genome of *E. tenella* H strain (a chromosome level genome to be published soon) using Hisat2 version 2.2.1 (Kim et al., 2019). The output SAM files were transformed into BAM files, which were then sorted and indexed. The sorted BAM files were then used for read count *via* htseq-count version 0.13.5 (Anders et al., 2015). Differentially expressed genes (DEGs) between groups were calculated by the R package DEseq2 (Love et al., 2014), and functional enrichment analysis (GO and KEGG) was performed using ClusterProfiler (v 4.0.5) (Wu et al., 2021). Gene expression with a fold change >2 or < −2 and an adjusted *p* < 0.01 was considered to be significantly differentially expressed. Transcripts per million (TPM) were calculated for each gene and used for clustered heatmap drawing.

### qPCR

To validate the RNA-seq data, we selected four DEGs for qPCR experiments. The cDNA samples were synthesized from DNase-treated RNAs employed in the RNA-Seq using TransScript One-Step gDNA Removal and cDNA Synthesis SuperMix (Transgen Biotech, Beijing). PCRs were performed on Roche LightCycler® 480 system using TransScript® II Green One-Step qRT-PCR SuperMix (TransGen Biotech, Beijing). For each sample, reactions were performed in three replicates. The primers are listed in the Supplementary Table 1. The expression of each gene was normalized to the reference gene glyceraldehyde 3-phosphate dehydrogenase (GAPDH) as reported previously (Hu et al., 2018). Unpaired two-tailed Student's *t*-tests were used for statistical analysis using GraphPad Prism® version 9.0.0 (GraphPad Software Inc., USA).

## Results

### Overview of the Sequencing Data and Gene Expression Patterns

**Figure 1:**
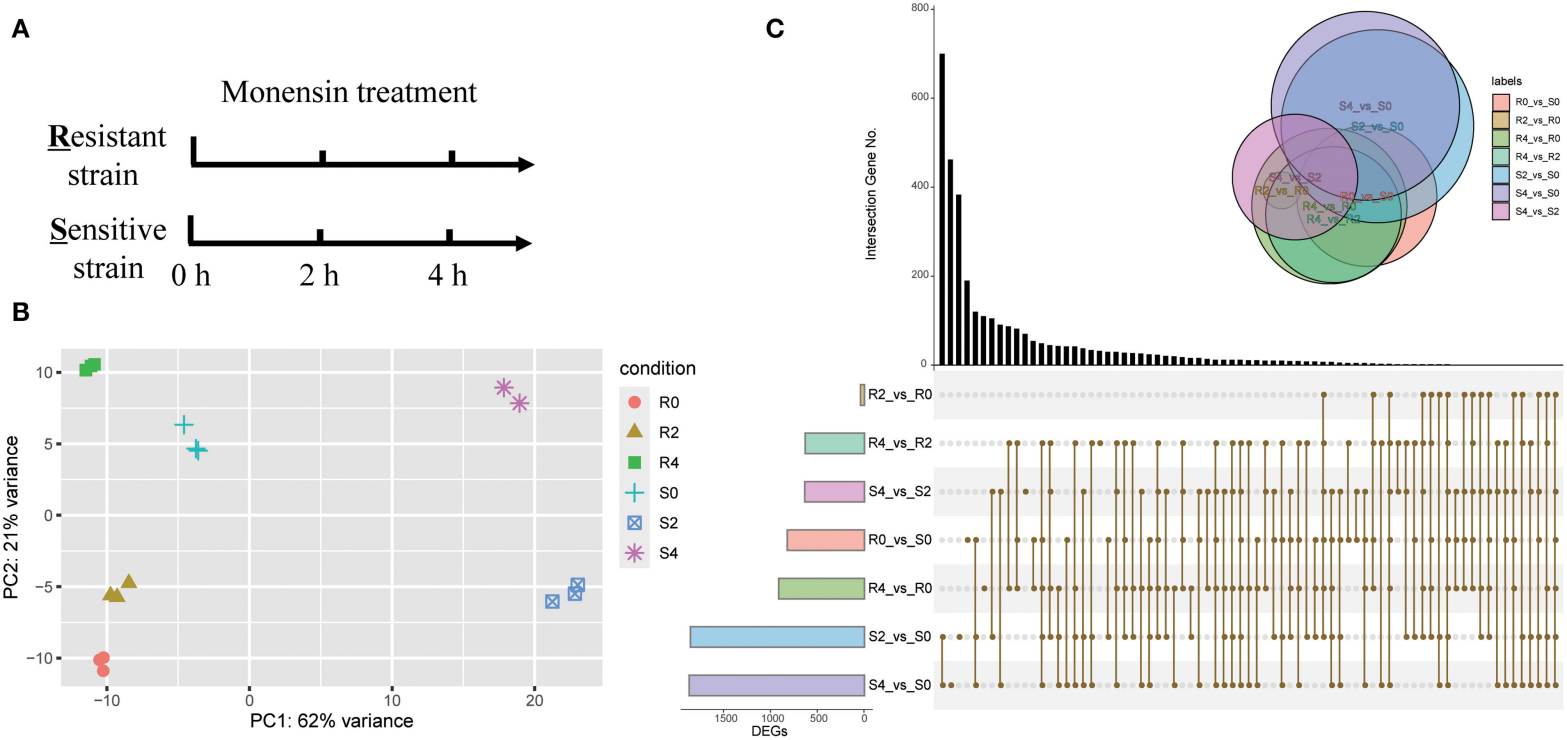
Overview of RNA-seq analysis. **(A)** Diagram showing the monensin treatment and sampling strategies. Fresh sporozoites of *E. tenella*-sensitive strain (S) and -resistant strain (R) were treated with 5 μg/ml monensin for 0, 2, and 4 h, respectively. Total RNAs were then subjected to RNA-seq. **(B)** PCA of all RNA-seq samples. **(C)** An upset-plot showing sets of differentially expressed genes after monensin treatment. Vertical bars show the number of intersecting genes between the comparison groups, denoted by the connected brown dots below the histogram. Horizontal bars show the size of DEGs between the comparison groups. The Venn diagram shows the size and overlapping situation of DEGs in the different groups.

As monensin primarily targets the sporozoite stage of *Eimeria*, we treated sporozoites of both resistant and sensitive strain with monensin *in vitro*. To gain insight into the early-stage gene expression landscape, sporozoites were treated for 0, 2, and 4 h and subsequently subjected to RNA-seq analysis (Figure 1A). A total of 410 million clean paired-end reads were generated and properly mapped to the reference genome (86.93–91.70% uniquely mapped), and samples could be grouped accordingly based on their background and monensin exposure (Figure 1B). To test the background of ETH-S and ETH-R, both heterozygous and homozygous SNPs were called out for all samples. It showed a relatively low level of mutants (SNP numbers ranging from 898 to 1,711, data not shown), suggesting that they have the same genetic background as the reference *E. tenella* Houghton strain. This study focuses on the transcriptome changes in the parasite in response to monensin, and the genetic variation and evolutionary trajectory during drug-resistant induction will be published elsewhere.

To evaluate the gene expression patterns after monensin exposure, DEGs of different groups were determined (Figure 1C, Supplementary Dataset 1). The comparison between S4 and S0, as well as S2 and S0, had the highest number of DEGs in all comparisons (1,862 and 1,848, respectively), and they also had the most specific DEGs (462 and 383, respectively). On the contrary, the comparison between R2 and R0 had the minimal number (31 DEGs, Figure 1C, Supplementary Dataset 2). These results revealed considerable changes in gene expression profile after 2 h of treatment in ETH-S, but the response only started in ETH-R. We also noted a lower number of DEGs between S4 and S2 (626 DEGs, Supplementary Dataset 3) than between S2 vs. S0 (Figure 1C, Supplementary Dataset 4), suggesting limited gene alteration upon extended exposure in the sensitive strain.

### Transcriptional Changes After Drug-Resistant Induction

**Figure 2:**
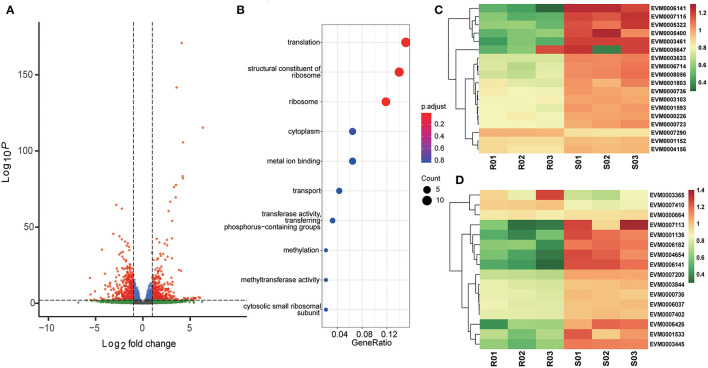
Differential gene expression after monensin induction. The *E. tenella-*sensitive Houghton strain (ETH-S) underwent 16 generations of positive selection under increasing monensin concentration and then generated a monensin-resistant *E. tenella* line (ETH-R). **(A)** Volcano plot showing the difference in gene expression between ETH-R and ETH-S at 0 h of monensin treatment. Genes with an absolute fold change >2 and adjusted *p* < 0.01 were considered significantly differentially expressed (red dots). **(B)** Gene ontology enrichment analysis of differentially expressed genes between ETH-R and ETH-S at 0 h of monensin treatment. Clustered heatmaps show differentially expressed genes associated with ribosomal proteins **(C)** and mitochondrial proteins **(D)** between ETH-R and ETH-S at 0 h of monensin treatment. For heatmap drawing, gene expression levels were transformed into normalized log_2_(TPM+1).

The ETH-S and ETH-R sporozoite transcriptomes were directly compared to detect the differences in gene expression after generations of selection. A total of 813 genes were significantly differentially expressed, including 424 upregulated and 389 downregulated (Figure 2A, Supplementary Dataset 5). Among the downregulated genes, those responsible for ribosomes (GO:0005840, adjusted *p* = 0.0681), translation (GO:0006412, adjusted *p* = 0.0681), and structural constituents of ribosomes (GO:0003735, adjusted *p* = 0.0681) were statistically significant (Figure 2B). These three GO terms shared the majority of their DEGs and were all associated with ribosomal proteins (Figure 2C), suggesting the downregulation of protein translation in the monensin-resistant strain. We also found that many proteins annotated as mitochondrial proteins were also downregulated in the resistant strain, including ribosomal proteins, tRNA synthetases, tRNA methyltransferase, cytochrome c, and cytochrome c oxidase subunit Vb (Figure 2D).

### Gene Alteration in *E. tenella* Strains in Response to Monensin Treatment

**Figure 3:**
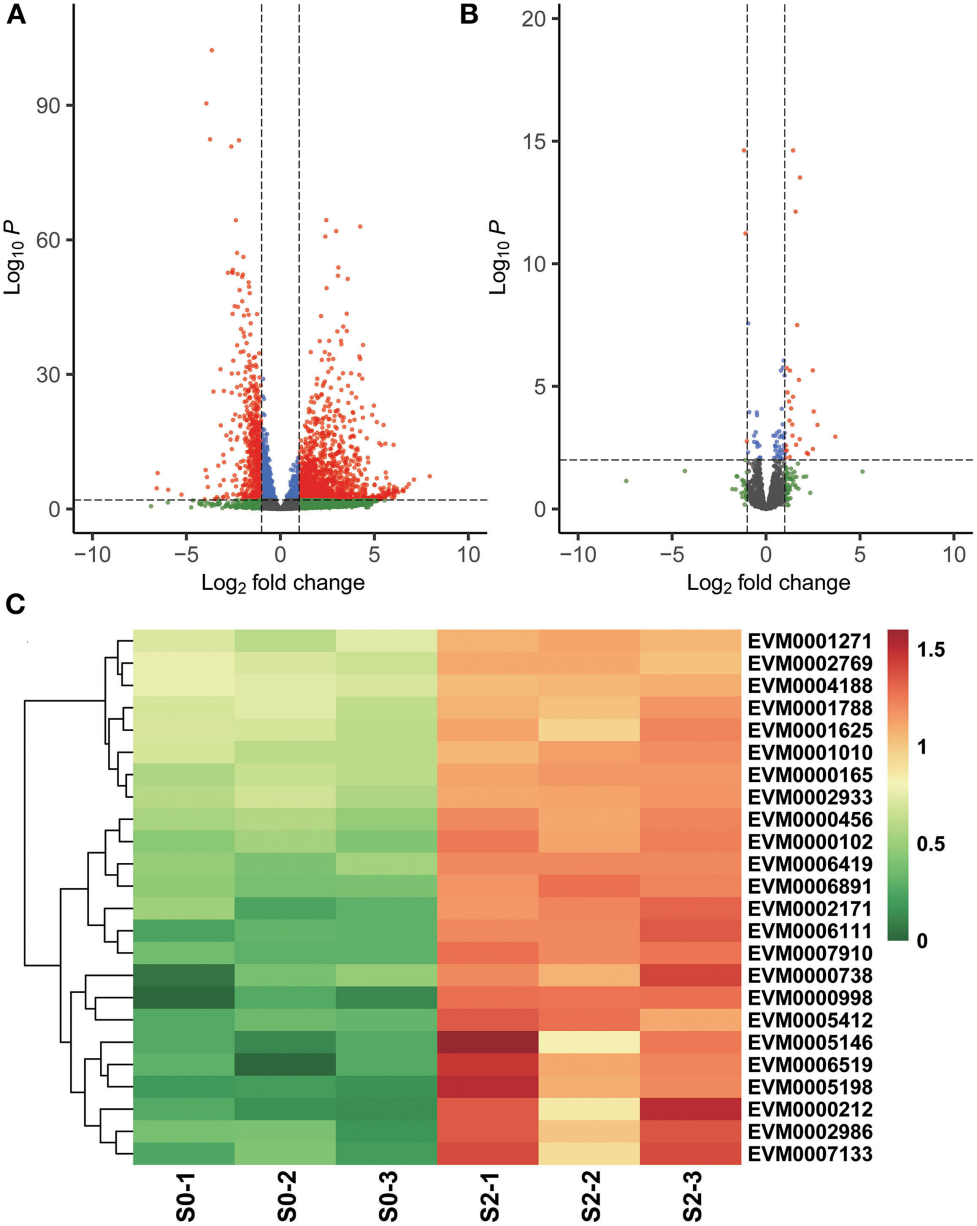
Differential gene expression after 2 h of monensin treatment. Volcano plots showing differentially expressed genes in the monensin-sensitive ETH-S strain **(A)** and the monensin-resistant ETH-R strain **(B)** after 2 h of monensin treatment. Genes with an absolute fold change >2 and adjusted *p* < 0.01 were considered significantly differentially expressed (red dots). **(C)** Heatmaps showing differentially expressed genes associated with ubiquitin and proteasome in the monensin-sensitive ETH-S strain after 2 h of monensin treatment. For heatmap drawing, gene expression levels were transformed into normalized log_2_(TPM+1).

After 2 h of monensin exposure, ETH-S and ETH-R parasites exhibited completely different scenarios. In the sensitive strain, a total of 1,848 DEGs were found after 2 h of treatment, of which 1,261 were upregulated and 587 were downregulated (Figure 3A). Among the upregulated genes, ubiquitin-dependent protein catabolic process (GO:0006511, *p* = 0.004841)-related DEGs were significantly enriched (Figure 3B), and more than 10 proteasome subunit proteins were also upregulated. These results suggest that protein degradation processes are activated in sensitive parasites after 2 h of treatment. Besides, many surface antigens and antioxidant proteins, such as thioredoxin and iron/manganese superoxide dismutase, were also upregulated (Supplementary Dataset 4). Regarding the resistant strain, only 31 DEGs were found after 2 h of monensin exposure, of which 28 were upregulated and 3 were downregulated. An ApiAP2 transcription factor EVM0000268 was 5.79-folds higher after drug treatment, which may account for the early gene regulation.

**Figure 4:**
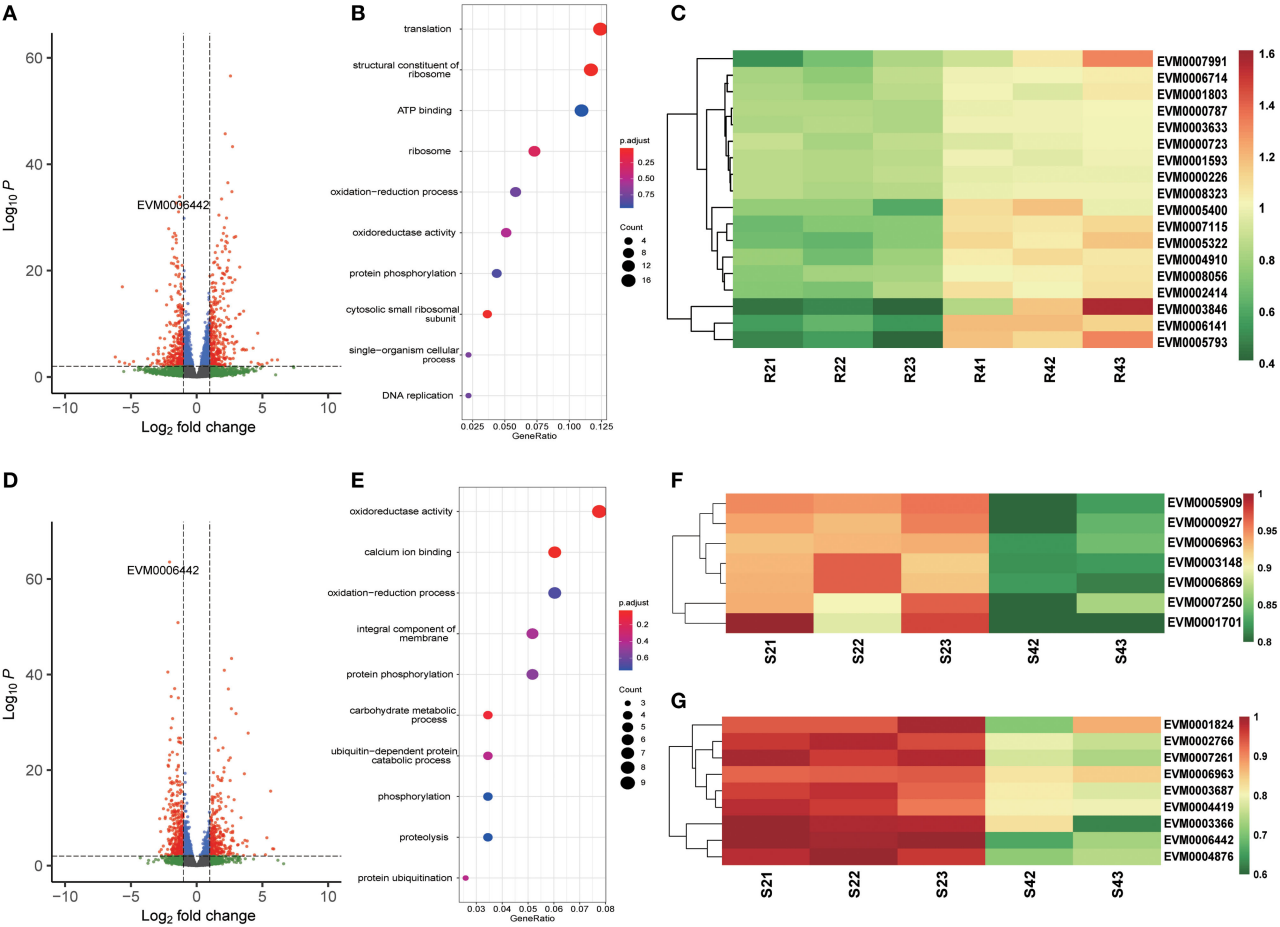
Differential gene expression after 4 h of monensin treatment. Volcano plots showing differentially expressed genes in the monensin-resistant ETH-R strain **(A)** and the monensin-sensitive ETH-S strain **(D)** after 4 h of monensin treatment. Genes with an absolute fold change >2 and adjusted *p* < 0.01 were considered significantly differentially expressed (red dots). **(B,E)** Gene ontology enrichment of differentially expressed genes in ETH-R **(B)** and ETH-S **(E)** after two additional hours of monensin treatment (4 vs. 2 h). **(C)** Heatmap showing differentially expressed genes associated with ribosomal proteins in the monensin-resistant ETH-R strain after two additional hours of monensin treatment. **(F,G)** Heatmaps showing differentially expressed genes associated with calcium ion binding proteins **(F)** and oxidoreductive proteins **(G)** in the monensin-sensitive ETH-S strain after two additional hours of monensin treatment. For heatmap drawing, gene expression levels were transformed into normalized log_2_(TPM+1).

**Figure 5:**
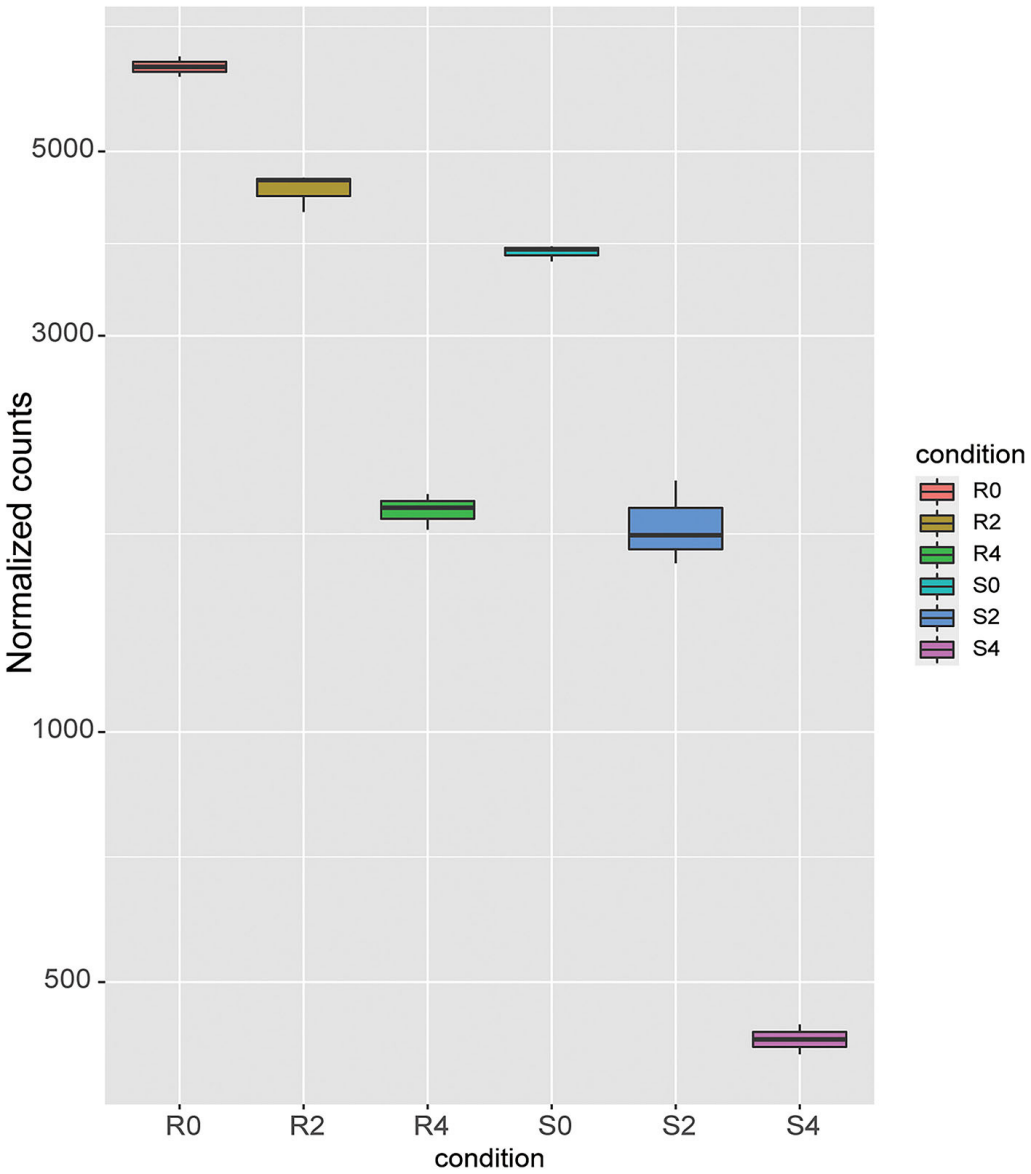
Gene expression level of FAD-dependent monooxygenases. Boxplot was drowned out by the R package DESeq2::plotCounts using normalized read counts.

After 4 h of monensin treatment, the monensin-resistant strain ETH-R showed more DEGs (621), of which 330 were upregulated and 291 were downregulated (Figure 4A, Supplementary Dataset 6). Translation-related genes (GO:0006412, adjusted *p*-value = 0.0000549) and structural constituents of ribosomes (GO:0003735, adjusted *p* = 0.0000549) were significantly enriched in the upregulated DEGs (Figures 4B,C). Compared to the 2-h treatment group, the sensitive ETH-S showed a total of 626 DEGs after 4 h exposure (Figure 4D), of which 292 were upregulated and 343 were downregulated. It can be noticed that the majority of gene expression alterations in the sensitive strain occurred in the first 2 h of treatment. Among the DEGs downregulated in ETH-S, genes involving in calcium ion binding (GO:0005509, adjusted *p* = 0.0269) and oxidoreductase activity (GO:0016491, adjusted *p* = 0.0347) were significantly enriched (Figures 4E–G). Among the DEGs, we noticed that a FAD-dependent monooxygenase (EVM0006442) was significantly upregulated in ETH-R and significantly downregulated after monensin treatment in both ETH-S and ETH-R. Interestingly, EVM0006442 maintained higher transcription level in ETH-R at the same time point of treatment (Figure 5, Supplementary Figure 1).

## Discussion

Ionophores are anticoccidial molecules commonly used in poultry industry, but resistance cases are frequently reported. To understand the molecular mechanism of drug resistance and the mode of action of ionophores, we induced a drug-resistant strain of *E. tenella* and compared the transcriptomes of the resistant strain and its sensitive ancestral strain in response to monensin treatment *in vitro*. We found that protein translation-related genes were significantly downregulated in the monensin-resistant strain and that gene expression was more susceptible in the ancestral strain in response to monensin treatment. The *E. tenella-*sensitive strain responded very rapidly to monensin treatment, with a total of 1,848 DEGs found after 2 h of exposure, nearly 60 times more than the resistant strain. Even after 2 more h of treatment, only 621 DEGs were found in the monensin-resistant strain. This result implies that the parasite genes of the resistant strain are slightly affected by the monensin treatment. Our data give an early gene expression landscape of *E. tenella* strains in response to monensin treatment and provide important information for further studies on ionophore resistance in coccidia.

Autophagy has been reported as a potential mechanism for monensin-induced parasite killing in *Toxoplasma* and *Eimeria*. Charvat and Arrizabalaga (2016) found that monensin treatment caused oxidative stress to the parasites, resulting in reduced mitochondrial membrane potential and morphological changes. The treatment also led to ATG8 re-localization from the cytoplasm to punctate autophagosomal structure parasite autophagy, suggesting that monensin induces autophagy in *T. gondii* (Lavine and Arrizabalaga, 2012). Monensin-induced autophagy and mitochondrial alterations (but not cell cycle arrest) can be blocked by the autophagy inhibitor 3-methyladenine (Lavine and Arrizabalaga, 2012). *In vitro* experiments on the sporozoites of *E. tenella* showed an elevated ratio of lipidated EtATG8 (form II) using western blotting with an anti-rEtATG8 antibody and also a concentrated localization of EtATG8 to the *E. tenella-*sensitive strain rather than to the resistant strain (Qi et al., 2020). In this study, a batch of proteasome subunits and ubiquitin proteins were significantly upregulated in the sensitive strain after 2 h of monensin exposure (Figure 3C), suggesting the activation of the proteasomal protein degradation process and monensin-mediated parasite killing. We also observed that several antioxidant genes, such as thioredoxin, were upregulated in the sensitive strain after 2 h of monensin exposure, possibly suggesting that the parasite is under oxidative stress caused by monensin. These results support the previous theory that monensin-mediated oxidative stress leads to mitochondrial alterations and parasite autophagy.

After 16 generations of induction, ETH-R showed the downregulated genes in the ribosome, translation, and structural constituents of the ribosome (Figures 2B,C). These pathways were all crucial for parasite protein translation, demonstrating the downregulation of protein synthesis activities in the drug-resistant strain. Besides, this may result in reduced fitness in this field, considering that parasites require a large number of proteins to complete their lifecycle, especially during the rapid amplification schizogony stage. However, these genes were then upregulated after 4 h of treatment in the *E. tenella-*resistant strain. Different results were observed in the *T. gondii* RH strain, cytoskeletal and transmembrane proteins were differently expressed in the monensin-resistant strain determined by proteomics (Thabet et al., 2018), and the other study showed that protein biosynthesis-related pathways (spliceosome, ribosome, and protein processing in the endoplasmic reticulum) were downregulated in *T. gondii* RH strain in response to 24 h of monensin treatment (Zhai et al., 2020). These results suggest that monensin treatment affects parasite protein synthesis and may have different effects on different parasite stages (e.g., sporozoites and tachyzoites).

Cytochrome P450 (CYP450) is a well-known drug target and is also responsible for the metabolic resistance of several drugs (Zhang et al., 2017; Weedall et al., 2019; Tchouakui et al., 2021). Both FAD-dependent monooxygenases and CYP450 are crucial microsomal proteins involved in the metabolism of non-nutritive foreign compounds (known as xenobiotics), and their main function is to add molecular oxygen to xenobiotics, making them soluble to ensure rapid excretion (Eswaramoorthy et al., 2006; Heine et al., 2018). In the malaria vector *Anopheles stephensi* (Vivekanandhan et al., 2021) and melon/cotton aphid *Aphis gossypii* (Chen et al., 2020), overexpression of the microsomal protein CYP450 contributes to the resistance of insecticides. In this study, we found that a FAD-dependent monooxygenase was significantly upregulated in the resistant strain and always maintained higher transcription level in the resistant strain at the same time point of treatment. We hypothesize that the higher expression level of FAD-dependent monooxygenase may increase the microsomal excretion capacity, which in turn promotes the metabolism of monensin in the parasite and makes it resistant to the drug. However, this should be validated by further experiments.

In general, we present here the comparative transcriptional profiles for sensitive and resistant strain of *E. tenella* in response to monensin treatment *in vitro*. This knowledge contributes to the understanding of the mode of action and mechanism of drug resistance to polyether ionophores in coccidia.

## Data Availability Statement

The datasets presented in this study can be found in online repositories. The names of the repository/repositories and accession number(s) can be found in the article/Supplementary Material.

## Ethics Statement

The animal study was reviewed and approved by the Administration Committee of Laboratory Animals in Guangxi University.

## Author Contributions

HZ: investigation, writing the original draft, and visualization. LZ: validation and visualization. HS: writing the original draft. XL: writing, reviewing, and editing the manuscript. XS: conceptualization and writing, reviewing, and editing the manuscript. DH: software, funding acquisition, and supervision. All authors read and approved the final manuscript.

## Funding

This work was supported by the Natural Science Foundation of Guangxi Zhuang Autonomous Region (Grant No. 2021GXNSFBA220057), the National Natural Science Foundation of China (Grant No. 32102694), and the Specific Research Project of Guangxi for Research Base and Talents (Grant No. AD21075028).

## Conflict of Interest

The authors declare that the research was conducted in the absence of any commercial or financial relationships that could be construed as a potential conflict of interest.

## Publisher's Note

All claims expressed in this article are solely those of the authors and do not necessarily represent those of their affiliated organizations, or those of the publisher, the editors and the reviewers. Any product that may be evaluated in this article, or claim that may be made by its manufacturer, is not guaranteed or endorsed by the publisher.

## Supplementary Material

Supplementary Figure 1 qPCR validation of differentially expressed genes. The expression of each gene was normalized to the EtGAPDH, and the unpaired two-tailed Student's t-tests were used for statistical analysis.

Supplementary Table 1 Primers used in qPCR experiments.

Supplementary Dataset 1 Eimeria tenella gene expression under monensin treatment for different times. Gene transcription level is represented by transcripts per million (TPM).

Supplementary Dataset 2 DEG analysis for the monensin-resistant ETH-R strain after 2h of monensin treatment.

Supplementary Dataset 3 DEG analysis for the monensin-sensitive ETH-S strain after 4h of monensin treatment.

Supplementary Dataset 4 DEG analysis for the monensin-sensitive ETH-S strain after 2h of monensin treatment.

Supplementary Dataset 5 DEG analysis between E. tenella monensin-resistant strain and its parental strain.

Supplementary Dataset 6 DEG analysis for the monensin-resistant ETH-R strain after 4h of monensin treatment.
