# Supplementary figures and images for: Early Transcriptional Response to Monensin in Sensitive and Resistant Strains of Eimeria tenella
Source: Front Microbiol. 2022 Jul 4;13:934153. doi: 10.3389/fmicb.2022.934153 (PMC9289555; doi:10.3389/fmicb.2022.934153)

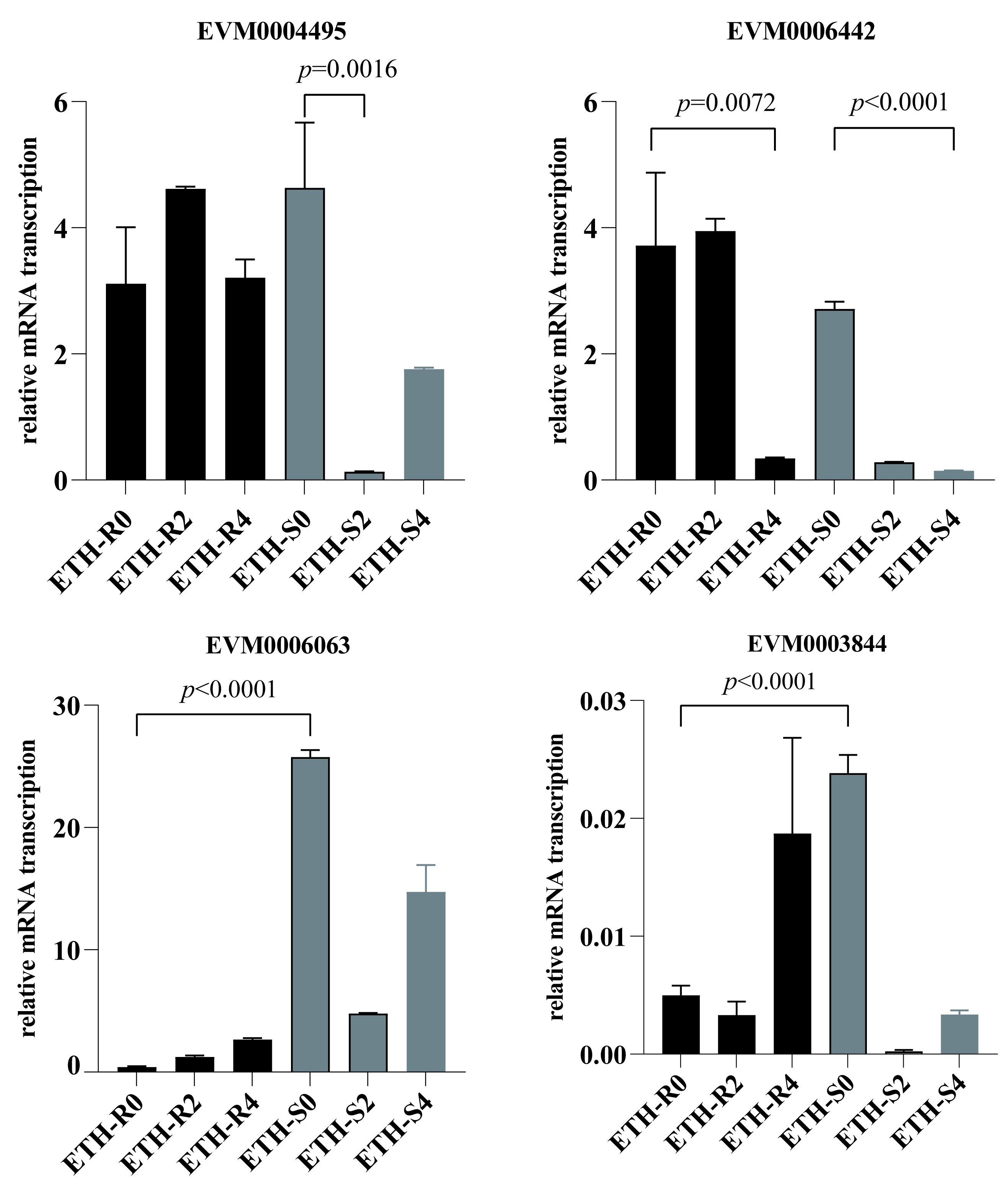

Supplement: Supplementary Figure 1 — qPCR validation of differentially expressed genes. The expression of each gene was normalized to the EtGAPDH, and the unpaired two-tailed Student's t-tests were used for statistical analysis. [file Image_1.TIF]
