# Supplementary material for: Early Transcriptional Response to Monensin in Sensitive and Resistant Strains of Eimeria tenella
Source: Front Microbiol. 2022 Jul 4;13:934153. doi: 10.3389/fmicb.2022.934153 (PMC9289555; doi:10.3389/fmicb.2022.934153)
Supplement: Supplementary Table 1 — Primers used in qPCR experiments. [file Table_1.DOCX]

**supplementary table 1：**Primers used in qPCR experiments.

| Primers | 5’-3’ |
| --- | --- |
| EVM0006442-F | ACTTCAGAGATTGTGCGGGG |
| EVM0006442-R | CACTTCGACCTTTGCGTTGG |
| EVM0004495-F | ACAGGAAGCGGATGTTCTCG |
| EVM0004495-R | ATTTTCCCCACCAAGCGGAT |
| EVM0006063-F | CCCAAGAAGAGCAAGCGAGA |
| EVM0006063-R | GGTGAGATCCCTTGCGTTGA |
| EVM0003844-F | CCACACAGTCAACAAAGGCG |
| EVM0003844-R | CCGCAAACACCATCTTCGTC |
| GAPDH-F | CATTGGGCGGTTGGTCTTCC |
| GAPDH-R | CCAGGATATCTGCCGTGGAC |
